# Turkish anime viewers’ approach to Japanese cuisine culture

**DOI:** 10.1186/s42779-021-00085-5

**Published:** 2021-06-21

**Authors:** Guliz Basaran, Serdar Sunnetcioglu

**Affiliations:** 1 Institution of Social Sciences, Department of Gastronomy and Culinary Arts, Canakkale Onsekiz Mart University, Canakkale, Turkey; 2 Faculty of Tourism, Department of Gastronomy and Culinary Arts, Canakkale Onsekiz Mart University, 1st Floor, Room Number: Z15, 17100 Canakkale, Turkey

**Keywords:** Anime, Japanese cuisine, Japanese culture, Food and beverage motivation

## Abstract

It is seen that anime, which is a Japanese animation type, with the food scenes in it, cause the curiosity and interest of the anime viewers from different cultures towards Japanese cuisine. It is thought to contribute to Japan in terms of gastronomy. Accordingly, although there are many promotional and marketing tools to spread the products of Japanese cuisine culture to the world, the idea that anime with a large global viewers also have a share in this promotion is the starting point of this study. Within this context, in this study, it was aimed to research how animes affect the perception of Japanese cuisine culture in individuals. At the same time, informations about the foods they prefer belonging to this culture have been obtained. Interview technique which is one of the qualitative research methods was used for data collection. The interview questions were sent to 31 anime viewers by selecting the snowball sample method, through Google Forms, and the answers were collected between 2 February 2021 and 16 February 2021. Descriptive and content analysis was applied to the obtained data. According to the results of the research, it is seen that animes have a serious contribution to the reflection of the Japanese cuisine culture. While the interesting scenes in the food-themed animes enable the viewers to try Japanese food, it has been determined that they encourage to buy Japanese cuisine products and use them at home. In general, it was observed that animes made nearly half of the participants curious about Japanese cuisine and animes directed them to experience it.

## Introduction

Animes are a type of animation that has been transferred to digital media, with the advancement of technology, from the comics called manga created by Japanese culture. In short, anime, which is fed by manga, meets the concept of “animation” in Japan. Reaching many people regardless of language, religion, race, and culture, these animated TV series have become a part of popular culture with their scenarios [1–4]. According to the researches on manga history, the change in style in Japanese daily life and art before and after the Meiji period. And the social, psychological, and cultural effects of American forces on Japanese culture during the American occupation after the Second World War are considered as historical processes that led to the development of manga culture and the reach to its contemporary meaning [5, 6]. The oldest known Japanese animation dates back to 1917. In the following years, many original Japanese cartoons were produced. Japanese animation developed in the 1960s, especially with the work of Osamu Tezuka [7, 8].

Since the 2000s, when the Internet made digital production and content-sharing accessible to the masses, contents from local communities have been starting to reach more and more people [9]. As an example of this, animes as native Japanese content spread as a major cultural export during the market expansion in the 1980s and 1990s. Anime is Japan’s main cultural export product [8, 10, 11]. Unlike Western comics and animations, manga and anime express the real life because it was developed from Japanese culture and targets adults, unlike Western animation [2, 12].

Cartoons emerge as one of the important visual elements that carry the narratives from the past to the present with cultural transfer. The most important examples of this are Walt Disney cartoons in the West and anime from Ghibli Studios in the East. Anime, unlike Walt Disney animation, includes topics that Western viewers are accustomed to see in live-action movies, such as romance, comedy, tragedy, adventure, even a kind of psychological review rarely found in Western movies [13]. That is why it has received global attention and targets not only children, but also teenagers and adults [10, 14].

Anime, as Japanese popular culture content, is attracting global customers by rapidly growing in popularity. This Japanization process, expressed as the worldwide spread of Japanese culture (karaoke, sushi, drama, anime, manga, Japanese management style, and industrial products) [15], clearly challenges Western hegemony and most existing theories about globalization. While in Japanization, Japanese anime is most commonly referred to, the effects of anime tourism can extend far beyond geographic boundaries [4]. Animes, one of the sub-branches of cinema, are defined as soft power by the Japanese government, as they are an instrument that has international impact [16, 17]. Thus, animes are the first and sometimes the only contact with Japan for many people.

With the growing foreign interest in anime, the Japanese government and tourism organizations use anime as an advertising mechanism to increase inbound tourism. It is also seen that anime is used in subway networks in Japan to increase domestic tourism [1]. Still, the increase of sites created with internationally renowned anime concepts and anime tours have played a role in the globalization of anime tourism [18]. Therefore, using anime as a way to promote cultural experiences in Japan to attract tourists is beneficial as it targets about 50 million people who are interested in Japanese anime [17, 19, 20].

The main motivation for anime-oriented travels are to visit places that have been used in anime series or movies [11]. Social interaction as a driving factor is a popular factor in the realization of leisure travel. The desire to experience the food culture and the social life seen in the anime that constitute the desire to visit Japan in terms of film tourism shows up as an important driving force [21].

In studies examining the relationship between anime and tourism, it is seen that terms such as “Otaku (anime fan) Tourism,” “Anime Tourism,” and “Anime Pilgrim” are used [18, 21, 22]. As a reflection of the imagination they created with their previous viewing experiences, visiting places where symbols and signs are concreted is considered a tourism activity that movie tourists want to experience [23, 24]. Based on this definition, the term “Anime Pilgrim” includes exploring and traveling places similar to the scenes in anime episodes [22, 24, 25]. As a result, it seems that manga and anime fans are the most important factor in the spread of Japanese popular culture, as they actively participate in this culture’s resources to be shared, consumed, produced, and reproduced [26].

It can be said that the fondness for Japanese culture generally began with anime [11]. It is stated that anime triggers the desire to learn Japanese on the viewers [13, 21]. For example, learning about chopsticks and how to use them through anime, people practice themselves as seen in the anime [17]. In many areas such as this example, elements of Japanese culture are transferred to viewers from other countries through anime.

Food is an important part of Japanese society. It is thought that there is an effect in Japanese food that inspires words such as unique and authentic, making us crave the “real one.” For this reason, Japanese cuisine is defined as “authentic” and “exotic” [15]. For most Europeans and Americans, for example, Japanese food of all kinds is seen as a relatively new experience [27]. Japanese cuisine, which also consists of healthy and sustainable products, contains the flavors offered by the geography it is located in [28]. Animes reveal that how appetizing the food can be in Japanese cuisine as well as the Japanese life, which they take as their subject, being neat, simple, and beautiful [6]. Many of the characters eat heartily, and the food in the Ghibli anime belongs to Japanese culture. For example, noodles are the most commonly consumed food we encounter. Then, traditional examples such as Sapporo beer, umeboshi (salted plums), okonomiyaki (a food between pancake and pizza), yakitori (grilled chicken shish), bento (lunchboxes), and Sosuke’s ham and noodle soup are the most common ones encountered in animes [1, 29]. Foods containing ramens, sushi, and rice balls are also items of other culinary culture frequently seen in animes [21].

Studies in national and international literature show that anime fans have approaches towards destination tourism [11, 17–22]. Many shared visuals which contains Japanese culture food that are made by fans in real life seen as in animes is being found on the digital platform. There are very few studies in the literature about anime and eating/drinking behaviors. Therefore, we try to enlighten on how animes affect the viewers to consume Japanese cuisine in this study.

## Methods

In this study, the interview form approach, which is one of the basic data collection tools of the interview method, was used to measure people’s perceptions [30, 31]. Because the interview form approach is a tool that consists of answering questions prepared by examining the subject of the research by using individual expressions and diversifying those according to people’s own style [31, 32], it was found appropriate for this study. At the same time, various information about a single subject was obtained from the participants by adhering to the research topic [31]. A structured interview form was prepared to collect data. This type of form consists of questions determined beforehand by the researcher. In this way, participants can give their answers without wasting time [32]. On the other hand, it is seen that rationality is high because there is little flexibility in the answers given to the structured forms [30]. Within the scope of this research, qualitative data were collected through the structured interview form prepared by reaching 31 participants between 2 February 2021 and 16 February 2021. While there are questions about demographic features in the first part of the form, there are questions about the research topic in the second part. The interviews, which were planned to be held face-to-face under normal conditions, were conducted online with the questionnaires created on Google Forms, in accordance with the COVID-19 measures. Anime viewers occur in the population. Participants in the study were reached using the snowball sample method. Snowball sample is defined as the sample type formed by the contact with other people after reaching the person who is thought to be a rich source of data for the research subject from the population [31, 32]. So in this study, first, we interviewed two anime fans, and then we asked them whether there are other people to recommend to interview. And data collection was continued this way. Beside snowball sampling, we share the interview form on the Instagram accounts created by Turkish anime fans (@animeturkiyetr and @animeturkiyeteam) to reach more people in order to collect more data. Finally, we stopped collecting when the data was saturated.

In this study, detailed evaluations were made by applying descriptive and content analysis. The data obtained according to the descriptive analysis were interpreted by the researcher by summarizing it in accordance with the themes prepared before according to the research questions. In content analysis, the notions and themes at the terminal points that are overlooked in descriptive analysis were discovered. In this way, the research results happen to be explained more logically [31]. Unlike quantitative researchs, various strategies were applied to ensure scientific acceptance in qualitative research. First of all, it was aimed to ensure the validity of the research by including the most comprehensive and satisfactory answers on the subject in the findings section. In qualitative research, direct quotations emerge as an important element to ensure the validity of the research. When necessary, presenting the data by directly quoting participant expressions increases the validity of the subject specificity [33]. Analyses made on data obtained by qualitative methods must have scientific credibility [32]. Assuming that other researchers also reached the same results with the strategies applied to the data, the reliability of the study is ensured. At the same time, the credibility, that is, validity, on the results were revealed by proving that the analysis was made independently of the researcher [31]. The validity-credibility strategy applied within the scope of this study is an expert review. In order to ensure the validity and credibility of the qualitative data collected for the research, reconciliation points were obtained by working with two researchers, apart from the researcher, who are experts in their fields [31]. In this way, it was aimed to provide credibility, consistency, and transmissibility by analyzing the data in a detailed and open manner.

## Results

Descriptive statistics about the participants are given in Table 1. The demographic features and the information about the frequency of watching anime of the participants are given in Table 1.

**Table 1:** Descriptive statistics

Descriptive statistics		N	%
**Gender of the participants**	Female	16	51.6%
	Male	15	48.4%
**Age of the participants**	18–24	24	77.4%
	25–34	7	22.6%
**Educational status of the participants**	Primary education	1	3.2%
	High school	11	35.5%
	Associate degree	2	6.45%
	Bachelor degree	13	41.95%
	Master degree	3	9.7%
	Doktoral degree	1	3.2%
**Profession of the participants**	Student	20	64.5%
	Other (engineer, game designer, research assistant, data scientist, economist, designer, teacher)	7	22.6%
	Not working	4	12.9%
**Marital status of the participants**	Single	29	93.5%
	Married	2	6.5%
**Monthly income status of the participants**	$180and under	19	61.29%
	$181–$300	5	16.12%
	$301–$540	3	9.6%
	$541 and above	5	16.12%
*Approximate value was calculated based on the exchange rate of 8.30 dollars by Turkish Liras.			
**Anime viewing frequency**			
**How many hours a week do you watch anime?**	1–2 h	8	25.80 %
	3–4 h	6	19.35%
	5–6 h	6	19.35%
	7 h and above	11	35.5%
	**Total**	**31**	**100**

### Results on the anime preference

As seen in Table 2, the participants became aware of the anime in various ways. It has been determined that the participants mostly met with anime through the social environment. On the other hand, as in the statements such as P20’s, “Being a child of the era when anime such as Versailles no Bara and Sailor Moon were broadcasted on television, provided me with this opportunity.” and P14’s “It popped up on my Instagram discovery and I liked it so I said let's see it and suddenly I was dragged into it.”, it is seen that people discovered animes through television (5) and social media (4). In addition, it can be said that animes are being discovered within the scope of language learning intention (1), as seen in P5’s statement, “When I started learning the Japanese language, I was looking for a source to practice the listening skill, anime was the funniest answer.”

**Table 2:** The ways of participants to be aware of animes

Categories	Frequency
**Social environment**	Friend recommendation (11) Cousin recommendation (2) School environment (1) Game environment (2)
**Media tools**	Social media (4) Internet (1) Television (5)
**Personal effort**	By oneself (4) Language learning intention (1)

When the participants were asked about the anime genres they are watching, 11 participants stated that they watch all kinds of anime. Answers received from other participants and their frequencies are shown in Table 3. When all these data are examined, besides Turkish genre names, anime genres were expressed in Japanese terms. It is seen that the most watched anime genre is the anime Shounen (9), which means “boy” in Japanese, where action and fight scenes are intensely processed. Then, it is seen that anime such as comedy (6), supernatural (4), science fiction (4), drama (4), and slice of life (3) are frequently watched. It is stated that besides the anime types frequently mentioned in Table 3, Tsundere, Ghibli, Seinen, Josei anime genres and mystery, love, and adventure-themed animes are also being watched by the participants.

**Table 3:** Watched anime types and names of food-themed anime

Categories	Frequency	
**Watched anime genres**	**Japanese anime genre names**	**Turkish anime genre names**
	Shounen (9) İsekai (3) Slice of life (3) Mecha (2) Shoujo (2)	Action (3) Supernatural (5) Comedy (6) Science fiction (4) Drama (4) Fantastic (2) Psychological (2) Thriller (2)
**Names of the watched food-themed animes**	Shokugeki no Souma (6) Food Wars (6) Bartender (2) Spirited Away (2) Naruto (4) Toriko (2) Ristorante Paradiso (1) Restaurant in Another World (1) Yume-Iro Pâtissière (1) Wakako-Zake (1) Moyashimon (1) Udon No Kuni No Kinino Kemari (1) Kakuriyo No Yadomeshi (1) Janggeum's Dream (1) Nagi No Asukara (1) Howls Moving Castle (1) Princess Mononoke (1)	

Twenty-one of the participants stated that they watch the food anime. Shokugeki no Souma (6) and Food Wars (6) are the animes coming to the forefront in the food-themed animes seen in 10 of the participants stated that they do not watch food animes in particular. In addition, two of these participants expressed that although they do not watch food-themed anime, food scenes are frequently featured in animes.

### Results about the effects of animes on participants

Participants stated that they watch the animes depending on the many factors animes represent. According to the data obtained, the factors that encourage watching animes were examined under 4 titles. These are detailed in Table 4 as psychological, social, cultural, and artistic factors.

**Table 4:** Factors that encourage watching anime

Categories	Frequency
**Psychological factors**	Improving mental state (2) Escaping from reality (5) Chill out (2)
**Social factors**	Entertaining (4) Pleasant (2)
**Cultural factors**	Japanese culture attracting attention (3) Learning Japanese (2) Contribution to the understanding of life (2)
**Artistic factors**	Interesting and unusual subjects (7) Style of drawings (8) Quality (2) Story and fiction (4) Creative (2) Inspiration (1) Music (2)

According to Table 4, it is observed that the participants tend to watch anime because of the interesting and extraordinary subjects of animes (7) and the different drawing style (8) as seen in P31’s statement: “Visual elements (drawing style, color selection), processing extraordinary subjects that would be difficult/impossible to film if desired.” At the same time, as in P19’s statement, “I feel far from the outside world. Putting all my troubles aside and watching it because it is the only activity I can do with pleasure” the feeling of escaping from reality (5) and its being entertaining (4) are considered among the factors that encourage people to watch anime. Also, with the support of animes to the cultural perspective that is considered as a contribution to the understanding of life (2) as in the statement of P20’s, “I am watching it because it helps me take the first step against cultural and historical events, supports me with an interesting and futuristic perspective about the future, assists my dream world, in short, contributes to my understanding of life”, it can be said that it is one of the factors leading animes to be watched.

Animes that affect the viewers in many ways are examined in 3 categories as socio-cultural, psychological, and mental effects, as seen in Table 5. As seen in the statement of P20 “It has creative and exciting effects on me as it still keeps my imagination energetic and guides me to perceive everything that I haven’t experienced, or the worlds I don't know. The animes and the episodes in animes on food also have an effect on me trying the recipes that I can make in the food and beverage field. I even think that students reacting cooking might be interested in the cooking after they watch the anime series (shougeki no Soyma, for example).”, animes are seen to have effects such as escaping from reality (5), providing motivation (4), being inspiring (3) by encouraging excitement (3) and creativity (2), and increasing imagination (2). At the same time, as in P13 stated, “It amuses, widens my horizon (inspiration). I learn about an another culture. I also learn our differences and in which point we are alike. Even though it is hearsay, somehow I learn the language. In addition, I experience a feeling of belonging to a worldview and a world other than the world I belong to.” alongside animes evaluated as pleasure and entertainment (3) factors, it can be said that animes have an effect on the participants in terms of being a tool for language learning (3) and getting information about different cultures (5).

**Table 5:** The effect of animes on participants

Categories	Frequency
**Socio-cultural effects**	Getting information about a different culture (5) Learning the language (3)
**Psychological effects**	Happiness (6) Feeling positive (4) Excitement (3) Relaxing (2) Removing the feeling of tiredness (1) Raising the morale (1) Pleasure and entertainment(3)
**Mental effects**	Escaping from reality (5) Motivation (4) Inspiring (3) Imagination (2) Creativity (2)

### Results on Japanese culinary culture

When the arousing curiosity of the anime on the participants towards the Japanese culture is examined, it is seen that 2 people answered both yes and no and 29 people gave answers such as “yes, absolutely yes, I agree, of course.” Yet, as it is seen in P20’s statement, “I absolutely agree. Aside from the food culture, by offering us so interesting, colorful and naive cultural festivals such as spiritual memorial days like Obon and cultural festivals like Tanabata, it definitely creates a dream to go and see there. In some way, I think they serve their cultural heritage in a great way,” it can be said that everything belonging to Japanese culture (14) and especially cultural foods (9) are reflected, and the desire to go and see Japan (5) is created in animes. At the same time, as in the statement of P15 “... such main factors like cultural elements, differences in language usage, palate difference, etc. shown in animes, attract attantion of people from other countries”, it is seen that animes are making viewers feel the cultural values as interesting (5).

When the participant were asked about the contribution of anime to the promotion of the Japanese cuisine culture, it is seen that most of the participants think that its contribution is great. By arousing curiosity (3) and interest (2) in Japanese cuisine on the participants, it is seen that animes encourage them to try (3) products as in P30 stated “It makes me curious. I got curious about the foods made in some of the animes that I watched and researched its recipe.” and also P31 stated “Animes introduced me to new types of foods, there are so many foods I saw in there that I cooked by finding the recipe.” At the same time, its being interesting along with curiosity lead to research on the cuisine. It can be said that animes have a positive effect (5) on the recognition of Japanese cuisine culture worldwide as in P14 stated “I think they are promoting them very well, thanks to the animes, I learned about Japanese cuisine culture.” It is also thought that animes contribute to the promotion (7) of Japanese cuisine as in P15 stated “It is a thing that the population of anime viewers are curious about the foods and try them, so I think it t contributes enough.” With this, animes are considered as a good marketing move in the recognition of Japanese cuisine culture in the world as in P2 stated “It is a successful marketing maneuver.”

All participants stated that different elements of Japanese culture are reflected in the animes they watched. As seen in Table 6, elements of popular culture are included in the animes in terms of preserving cuisine culture and values as well as the general knowledge of Japanese culture. Considering the frequency rates, it is seen that elements belonging to food culture (18) are frequently encountered in animes. In animes, respect and etiquette (7), ways of living social life (6) and history and mythological (7) narratives come to the forefront when looking at the other cultural value elements reflected in the animes as in the expression of P5 “It is possible to encounter many Japanese cultural elements from cuisine to religious ceremonies in animes. Many different details such as discipline in schools, social respect rules, superstitions can be examined.” Dressing style (6), traditional (5) festivals, relationships, and communication style (5) are among the cultural elements that are frequently encountered in animes and reflected to the viewers as seen in the statements of P31 “High school life, the importance of the bond of friendship, etiquette, traditional clothing and food" and P20 “Many values are given through animes such as Kimono varieties, dressing rules, big and small festivals, especially Tanabata and Obon, house-palace and street food culture, drinking culture, interpersonal relations, temples and gods, dances related to the religious field, and superior-subordinate relationships. That, I think anime artists and publishers using these as a kind of attraction element.”

**Table 6:** Japanese cultural values in animes

Categories	Frequency
**Cuisine culture values**	Food culture (18) Table manners (2)
**General knowledge values**	Respect and etiquette (7) Form of adress (2) Ways of living social life (6) Relations and communication (5) School life (5) Dressing style (6)
**Popular culture values**	Importance of religion and rituals (5) History and mythology (7) Preservation of culture (3) Traditionalism (5)

When the effects of eating and drinking rituals, which are among the values of the cuisine culture reflected in the animes, on the viewers were examined, it was found that they have an intensely interesting (5) and appetizing (5) effect. Also, it was said that as in the expression of P1, “I think they preserve some of the culture from the past. It is very interesting,” food scenes in the animes reflect the Japanese cuisine culture in the best manner by protecting (3) it. Participants touched on the chewing loudly habit (2) as in P20 stated “A society that has struggled with famine for many years and is still grateful to eat even a bowl of rice on its table, is one of the biggest things that I like. The only thing I find strange is that they slurp their mouth a lot and get big bites” alongside the fact that Japanese eating/drinking rituals are primarily different from other cultures (4) as in P13 stated “Some food processing and consumption styles can sometimes seem extreme or different even for people who have adopted their culture. I can say that Japanese food culture is different for each individual because it is a sector (food sector) that addresses the diversification of traditional and popular eating and drinking culture and the interests of different groups." It is seen that one of the most prominent features about rituals that they have respect for the food and the dinner table (2) by setting extravagant tables (5) with a carefully prepared feast consistency as in the statement of P5 “I find it a bit artistic. I have observed that the meals are prepared with care and attention is paid to the service to make it particularly attractive. I'm not sure if Japanese culture is the same, but to many other cuisines I could compare, it is a feast to my eyes.”

When asked about the similarities of the values of Japanese cuisine culture in the animes with the Turkish traditions, some of the participants mentioned about similar cuisines such as the use of the spices (3), meat consumption (2), rice consumption (3), soup made in the kitchen, stew foods, pickles used with noodles, pickles, and Turkish wraps—liken to sushi and onigiri. Expressing the similarities in table manners, 3 participants stated that waiting for adults at the table to eat is a common feature with Turks. Five participants touched on the similarity about the importance of respect at the table as in P15 stated “It is very evident in the richness of their meals that the elders prepare the meals with great effort and care home cooking, and it has similar characteristics with Turkish culture in this respect.” With the statement of P30, “The importance of the food and the dinner table in daily life, the care shown to it, the preparation of the meal and eating are often a home / family ritual” it is emphasized that eating at home by setting a table is a common feature. At the same time, while mentioning the similarity of the rituals during the meal as in P31 stated “Waiting for everyone to sit at the table, expressions of gratitude used before starting the meal and after the meal is finished.” One of the participants stated that the feature of showing honor to the guests is one of the similarities between Turkish and Japanese culinary cultures. On the similarity of food culture, 2 participants talked about the variety of food being a lot in both cultures. In addition to all these, the similarity between cultures is also stated in terms of the presence of women in the kitchen as in P2 stated “Being a mother in the kitchen in general is the result of the patriarchal life in its history. It is the same, in our culture, with the logic of respect for blessings and avoiding waste…” On the other hand, 7 participants stated that there is no similarity. One of these participants stated that there is no similarity since it is a cuisine with a high concentration of seafood. Three participants stated that they do not know much about the cuisine.

When the effects of animes on eating/drinking behaviors are evaluated as frequency according to the responses given by the participants, it is seen that the use of chopsticks (3) increased and there is a preference for eating noodles (4) in terms of easiness. In addition, viewers have stated that they make onigiri and ramen at their homes, consume dried seaweed if they find it, and order sushi from outside. It is seen that animes have effects on the preparation of the food at home as seen in animes (7) and consumpiton of green tea (2) as it is seen in P20’s statement “Like any anime viewer, I started with ready-made ramen and then learned how to do it and did it myself. I tried the recipes as long as I could find the ingredients. The best thing for me is that I stopped consuming bread and eat rice instead. My attitude towards green tea has changed and I have been consuming more. I started to be attentive to cook the ingredients less,” and the desire to eat something (2) when they see food scenes and the increase in knowledge about Japanese food (2) as it is seen in P3’s statement “I already love Japanese cuisine, but my knowledge and desire to eat increase because of the animes.” Sixteen people stated that animes have no effect them about Japanese cuisine.

The effects of animes on your preference of Japanese culinary products: 5 participants stated that they have no effect. Eight people stated that they have a lot of positive effects. Two of them mentioned that the drawings are beautiful. It is seen that because the food is eaten heartily in animes, alongside obtaining information about Japanese cuisine, 10 participants are in tendency to taste and try the food with curiosity as in P14 stated “When it is told about it in animes, it arouses the desire, the curiosity in me and makes me look into it.” The rest of the participants expressed that they brought products such as omurice, onigiri (2), dumpling, ramen, and green tea into their lives by obtaining ingredients such as soy sauce (4), sesame oil (2), pickled ginger, and rice vinegar as it is seen in the statements of P13 “To put it simply, Omurice is a popular food. To do this at home (in the most accurate way), it is necessary to provide some products. Rice vinegar, soy sauce and sesame oil and etc.” and P16 “I have started using soy sauce, sesame oil, pickled ginger.”

**Fig. 1:**
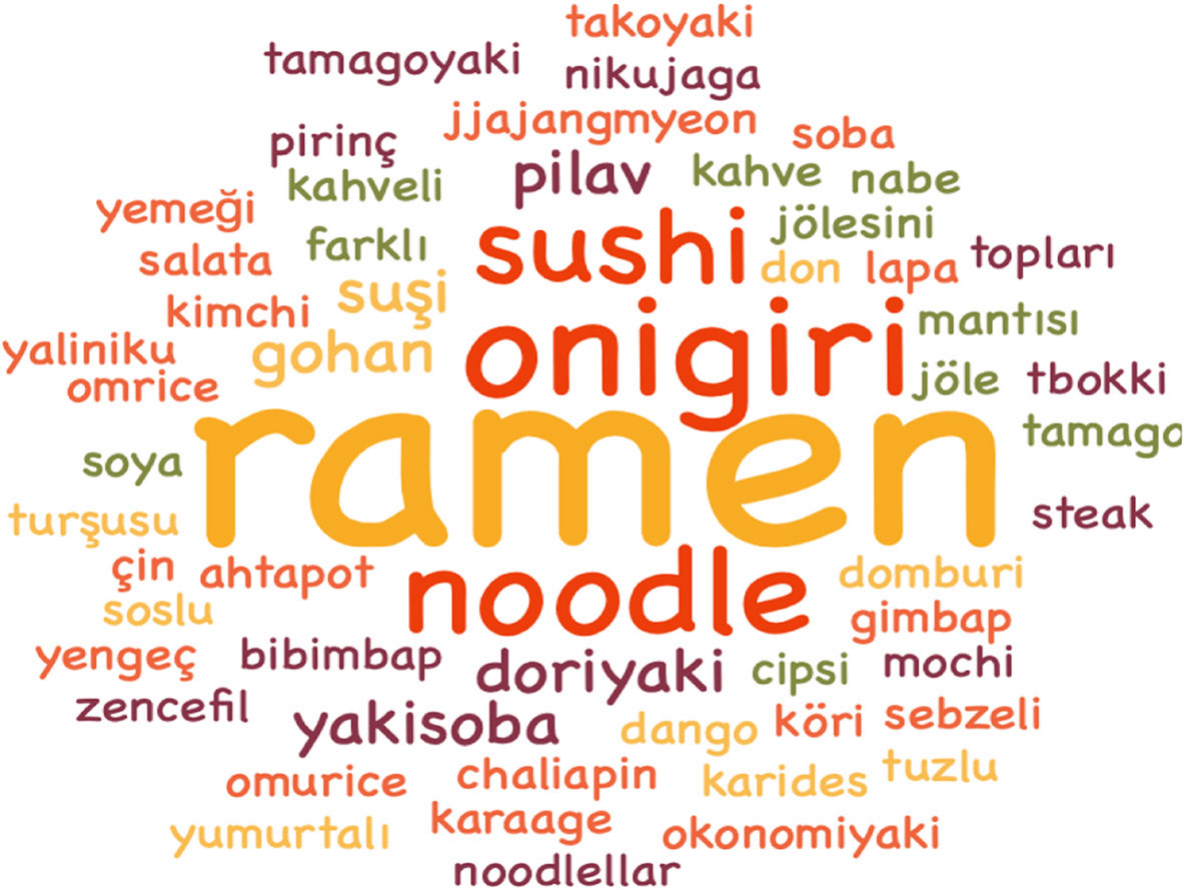
Foods tried or cooked at home with the effect of animes

Participants were asked about the foods they saw and tried in the food scenes after watching anime. Seven participants stated that they did not try any of these foods at home. Statements of the remaining participants are included in Table 7. At the same time, while analyzing the answers given by the participants to this question, meaningless words were ignored and a word cloud was created on the tried culinary products as seen in Fig. 1.

**Table 7:** Food and products in animes

Categories	Frequency	
**Foods made after being seen in animes**	Ramen (15) Onigiri (8) Sushi (7) Noodle (7)	Omurice (2) Dorayaki (2) Yakisoba (2) Coffee jelly (2)
**The most preferred products seen in animes**	Noodle (10) Ramen (9) Onigiri (5) Sushi (5) Takoyaki (3)	Yakisoba (2) Green tea (2) Soy sauce (6) Sesame oil (2) Chopstick (3)

As seen in Table 7, the most tried product was ramen (15) and onigiri (8). Apart from the items in Table 7, the participants included trying Japanese culinary products such as dried seaweed, rice porridge, rice with eggs, rice balls, salad with soy sauce and vegetable, dumplings, gimbap, bibimbap, jjajangmyeon, tbokki, crab, octopus, shrimp chips, green tea, mochi, kaage, domburi, kimchi, tamagoyaki, nikujaga, yaliniku, Chaliapin Steak Don, Tamago Gohan, pickled ginger, curry dishes, dango okonomiyaki, and nabe.

**Fig. 2:**
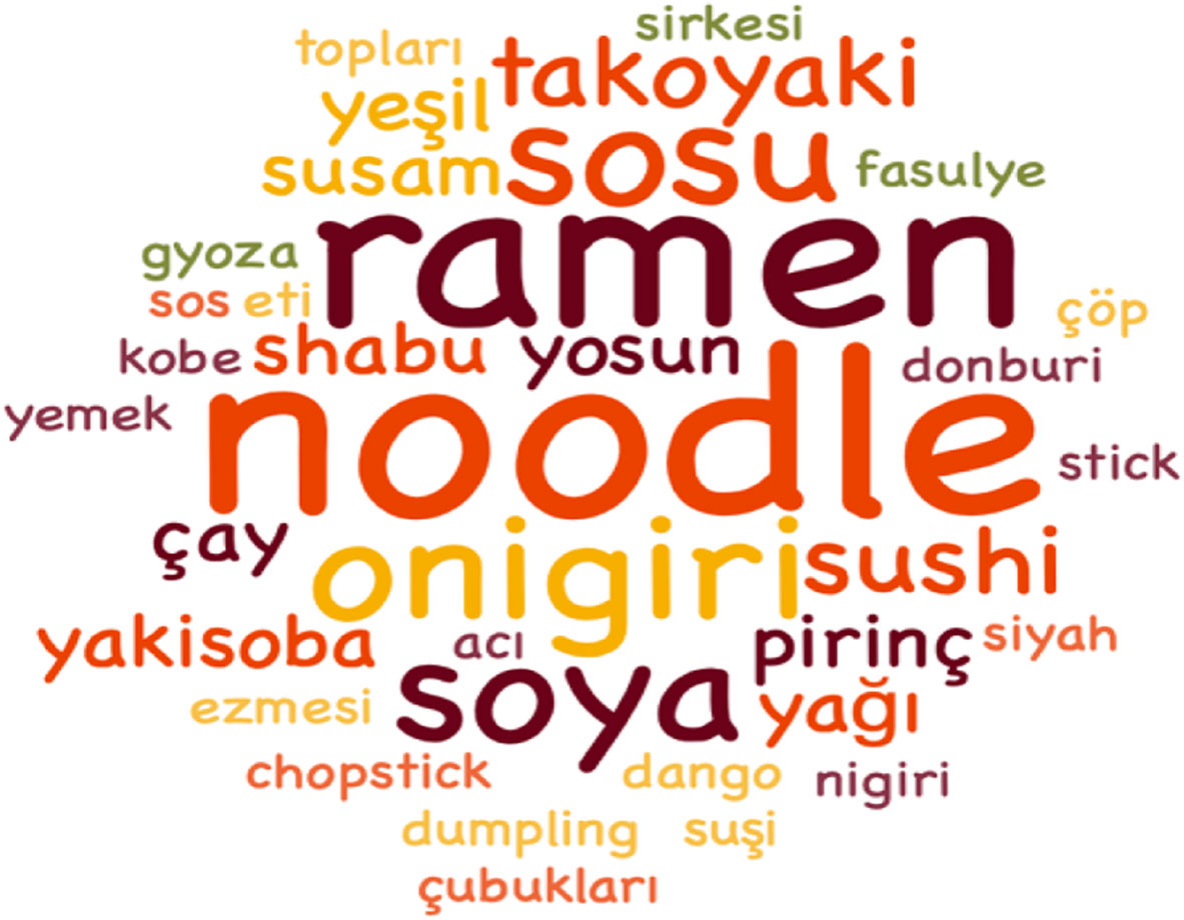
The most preferred Japanese culinary products with the effect of animes

As seen in Fig. 2, a word cloud was created with the culinary products that stand out in the answers given by the participants, who are asked about the products they see and prefer in the animes. At the same time, they stated that the products were mostly noodle (10), ramen (9), onigiri (5), and sushi (5), as seen in Table 7. Apart from foods, it is seen that there are participants prefering soy sauce (6) and sesame oil (2) as their culinary product after watching animes. Apart from that, it is determined that the participants who stated that they prefer to use bowls and chopsticks (3) are also affected by the animes in the use of rice vinegar, hot sauce, and spices. In addition to all these, it is seen that some participants prefer the consumption of gyoza (dumplings), kobe (steak), shabu shabu (thinly sliced meat and vegetables in boiling water), black bean paste, rice ball, dango (Japanese dessert), and donburi (rice bowl).

Finally, 10 of the participants, who were asked about other elements they want to mention about the food-themed animes, preferred not to state anything extra, and three people said that they do not watch food-themed animes. While 4 of the remaining participants emphasized them appetizing as in P7 stated “When watched, it gives a feeling of happiness and hunger”, three of them stated interesting, pleasant and beautiful, and two participants stated that they are entertaining. Similarly to P27’s statemant as “I think they have an important role in promoting Japanese cuisine and world cuisine”, 6 of the participants mentioned that it is a very useful tool in promoting and informing about Japanese cuisine culture. In addition, animes are seen as a source of motivation as in P28 stated “I am someone who wants to be a chef, it excites me to learn something as I watch.”

## Discussion and conclusion

The development of technology demonstrate its effect on many areas. One of them is the animation industry. This process, which started with the creation of Walt Disney cartoons in the West, stands out in the East with anime, which is the name of manga on the digital platform. Animes, which have many viewers on a global scale, are considered as examples presented interestingly of both their subjects and the art of animation. Animes and the culture they reflect are seen as Japan’s prominent export product in cultural dimension by rapidly gaining momentum since the 1990s. In this study, the perception and the effect of Japanese cuisine culture reflected with food scenes in animes and food-themed animes on the viewers is examined.

According to this research, although it is revealed that most of the participants are aware of the animes with the recommendations from their social circles, it is concluded that social media platforms facilitate the discovery thanks to the developing Internet network and media tools such as television which also cause people to meet animes. Although it is seen that the anime viewers in this study are mostly individuals under the age of 25, animes target a wide age range with many subjects it covers. Considering that the participants consist of a young audience, it is concluded that the animes named Shounen, which are action-themed on the basis of the subject and the cycle of events revolving around the young male character, are the most preferred. At the same time, with this study, it is determined that there are many anime genres created with advanced script and drawing techniques, and it is seen that the preference of themes such as science fiction, fantasy, and supernatural other than action come to the forefront.

The findings obtained in this study are similar to the study of Steine (2019), in which findings show that people tend to watch anime because it contains many elements belonging to the structure of Japanese culture, which is seen as different from most cultures, and contributes to the recognition of the culture [11]. It is determined that especially the preservation of the culture with the rituals and history in the tradition of Japanese culture are found to be interesting by most viewers. In parallel with the results of this study, in the study conducted by Kanama and Nakazawa (2017), it is concluded that traditionalism is preserved in the production of materials belonging to Japanese cuisine culture and that only today’s technologies are up-to-date in terms of product packaging and design [34].

It has been determined that the individuals, together with the animes, improve their mood and have fun time by establishing a psychological reality away from the stress of daily life. When the socio-cultural effect examined, it is seen that thanks to animes, the desire of the viewers to learn Japanese language is also determined as a result of this research, as in the study of Fukunaga (2006) [13]. Likewise, similar to the results obtained in Yıldırım et al.(2017)’s study in which the motivation for traveling to Japan thanks to the animes is discussed, it is determined with this research that the curiosity towards culture triggers the desire to visit the country [21].

In the study of Fanelli and Nocera (2018) in which the approaches to the preference of Japanese food examined, it is concluded that the urge to experience cultural values, which people see culturally different, contributes to the preference of Japanese food [27]. Likewise, in addition to the detailed visualization of the Japanese culture traditions reflected in the animes, the results of this study is similar to Öztekin (2008)’s study in which it is stated that the attractive appearance of the food in Japanese cuisine created by animation techniques and effects impresses the viewers [6]. In line with the data obtained, individuals find the food scenes in the animes quite appetizing and by seeing foods in the animes, they develop a consumer behavior by trying the culinary products. Therefore, it can be said that anime has an effect on the eating and drinking behavior of the participants.

Similar to the results of the food of Japanese cuisine, in the study of Yıldırım et al., (2017) noodles, ramen, sushi, and rice-containing foods, that are frequently seen in animes and arouse curiosity in the viewers, were identified as culinary products and unlike the other study, onigiri become the prominent type of food in this study [21]. In addition, it is determined that the participants use culinary products such as sesame oil and soy sauce in their foods, and it is observed that they increase the variety of spices and the amount of bitterness with the effect of animes. In general, seeings the attitude of the viewers towards purchasing the Japanese cuisine and cultural products reflected by the animes, it is concluded similarly also in the study of Tey, Arsil, Liew, Teoh, and Terano [35], that the interest and the curiosity in Japanese culture and traditions, in addition to its healthy content and functional food, contribute to the purchase of Japanese culinary products [36]. As Rachman (2017) stated in his research that the use of chopsticks, which is the symbol of Japanese cuisine culture in addition to food and animes, has become widespread, also revealed within this study, and in the same time it is concluded that bowls are preferred instead of flat plates while eating [17].

Although it is stated that there is no similarity with Turkish cuisine due to the fact that sea products are intensely used and the products are being cooked and consumed with different techniques, Japanese cuisine was found to be similar to Turkish traditions by the participants with the cultural values reflected in the animes. Expressions such as respect in the family, table manners, the habit of eating together, the dominance of a female figure in the kitchen, and the feeling of gratitude for the meal are among the prominent results of the research.

In findings, nearly half of the participants said that animes have no effect to them about Japanese cuisine. This may be because these participants prefer to watch animes to wasting their times and also they are not interested in cooking and trying Japanese foods.

Animes, which reflect the tradition of Japanese culture with advanced drawing and techniques in a technological context, are considered as tools that have many effects on their viewers. These animations, which have become a widespread trend, introduce Japanese culture to people with topics that appeal to people of all ages and make them interesting. Within the scope of this study, Japanese cuisine culture is discussed, and thanks to the animes, it is seen that many people become acquainted with various foods and culinary products by doing research on this culture. At the same time, measuring the effect of animes on the recognition of the cuisine culture in the field of gastronomy, it is revealed as a guiding element on consumer behavior. With this research, it is provided to examine in detail the feelings and thoughts created by the animes in people based on Japanese cuisine. The limitation of this study concerns the generalization. However, since the study is a qualitative research, the main purpose is not to generalize, but to examine participant experiences in depth. The other issue is participants’ ages. In the study, generally young participants were interviewed, but in another study conducted in Turkey, it was observed that the sample age range was similar [26].

In the future researches, it can be investigated with quantitative data that whether animes have an effect on consumers about experiencing Japanese cuisine by using existing scales in the literature or developing a new scale. Apart from this, it is thought that it can be researched in different studies that whether anime or other ethnic film elements affect people’s ethnic restaurant preferences.

## Data Availability

All data generated or analyzed during this study are included in this published article.
